# Intricacies in the surgical management of appendiceal mucinous cystadenoma: a case report and review of the literature

**DOI:** 10.1186/1752-1947-4-129

**Published:** 2010-05-05

**Authors:** Muhammad R Khan, Rashida Ahmed, Taimur Saleem

**Affiliations:** 1 Section of General Surgery, Department of Surgery, Aga Khan University, Stadium Road, Karachi 74800, Pakistan; 2 Department of Pathology, Aga Khan University, Stadium Road, Karachi 74800, Pakistan; 3 Medical College, Aga Khan University, Stadium Road, Karachi 74800, Pakistan

## Abstract

**Introduction:**

Mucinous cystadenoma is a type of mucocele of the appendix that is rarely encountered in clinical practice. Dogmatic consensus on the optimal surgical modus operandi of appendicular mucocele is lacking in the literature and this remains a subject of controversy. There is little agreement with regard to the best procedure (right hemicolectomy versus appendectomy) or the best surgical approach (laparoscopic versus laparotomy).

**Case presentation:**

We report the case of a 70-year-old Asian woman from Karachi who presented with pain in the right iliac fossa for 15 days. On physical examination, a mobile and firm mass was palpable in the right iliac fossa. A colonoscopy was performed which showed external compression of the cecum. A biopsy of the mucosa was normal. Computed tomography scan showed a mucocele of the appendix with minimal periappendiceal fat stranding. She underwent an initial diagnostic laparoscopy to evaluate any mucin spillage in the peritoneal cavity. Once no spillage was identified, an open appendectomy was then performed. Intra-operatively, a frozen section of the appendiceal sample was sent to ascertain the need for an extension of surgery to a right hemicolectomy. Absence of any malignancy on the frozen section obviated the need for a surgical extension. The final histopathological examination showed a mucinous cystadenoma of the appendix. The patient was symptom-free at one year after surgery.

**Conclusion:**

It is important to distinguish between mucinous cystadenomas and mucinous cystadenocarcinomas. However, this distinction remains elusive in the pre-operative setting. A simple appendectomy using an intra-operative frozen section appears to be a reasonable surgical approach for selected cases with an intact mucocele of the appendix. However, long-term follow-up is warranted in such patients to evaluate the risks of using this approach.

## Introduction

Mucinous cystadenoma, a type of appendiceal mucocele, is a rare clinical entity [1]. Mucoceles of the appendix account for only about 0.3% of appendix specimens, with mucinous cystadenomas being the most commonly encountered appendiceal mucoceles [2]. Mucoceles that are less than 2 cm in diameter are usually simple retention cysts, while hyperplastic epithelium, cystadenoma and cystadenocarcinoma are more likely to be greater than 2 cm [3]. In a review of 2660 appendectomy specimens, mucinous cystadenoma was reported in 0.6% of the cases [4].

A consensus on the optimal management of this condition has not yet been reached. Some authors have advocated the use of appendectomy alone for appendicular mucoceles while others support the undertaking of a right hemicolectomy. We report the case of a 70-year-old woman who underwent an open appendectomy and was found to have mucinous cystadenoma of the appendix. In addition, we have also reviewed the indications outlined in the literature for various surgical approaches for appendicular mucoceles.

## Case presentation

A 70-year-old Asian woman from Karachi presented with a history of pain in the right iliac fossa (RIF) for 15 days. The pain was pricking in nature, moderate in intensity and non-radiating. She also had a six-month history of a dragging sensation in her lower abdomen. There was no history of bleeding per rectum or documented weight loss. She was hemodynamically stable at the time of presentation. A physical examination showed tenderness and a firm, mobile mass of 5 × 2 cm in the RIF. Bowel sounds were audible and rebound tenderness could not be elicited. Her laboratory work-up including complete blood count, serum creatinine and liver function tests were all within normal limits.

**Figure 1:**
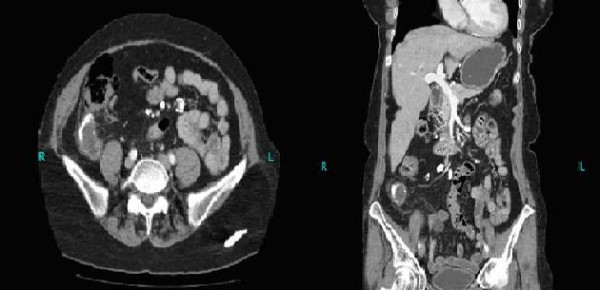
**Computed tomography scan showing calcified appendiceal mucocele and calcified mesenteric lymph nodes**.

She next underwent upper and lower gastrointestinal endoscopies which revealed an esophageal diverticulum, hiatal hernia, mild chronic *Helicobacter pylori *gastritis and evidence of external compression of the cecum. The mucosal biopsies from her cecum were reported as benign. A computed tomography (CT) scan of the abdomen and pelvis was subsequently obtained which showed a calcified mucocele of the appendix along with multiple calcified mesenteric lymph nodes. Minimal periappendiceal fat infiltration was also seen (Figure 1).

She was electively admitted and an informed consent for appendectomy or possible right hemicolectomy was obtained. She underwent an initial diagnostic laparoscopy to evaluate any mucin spillage or seeding of the peritoneal cavity before surgical intervention, because any such finding could mandate a more extensive procedure. Once no spillage was identified, we proceeded with open surgery using a limited transverse muscle cutting incision. The appendix was carefully dissected and removed. Intra-operative findings included an appendicular mass which was most likely a previously perforated appendix along the lateral abdominal wall. The lateral abdominal wall peritoneum was excised with the appendix to prevent any spillage of appendiceal contents into the abdominal cavity. The base of the appendix was fixed using absorbable transfixation sutures.

**Figure 2:**
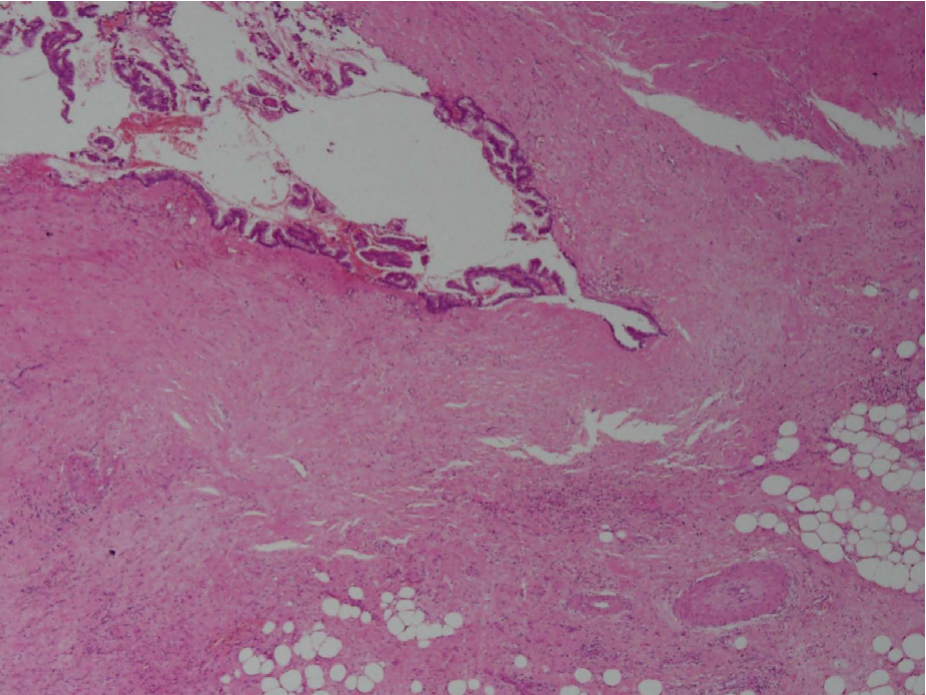
**Mucin secreting epithelium lining the appendix under magnification 4 × 10 (hematoxylin and eosin stain)**.

**Figure 3:**
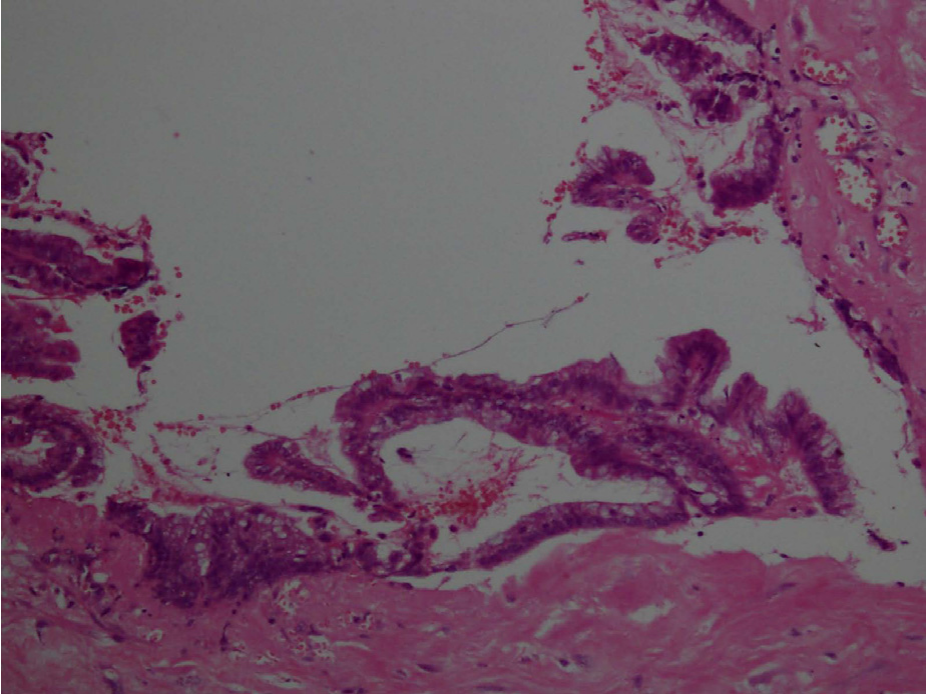
**The same field under a higher magnification of 20 × 10 (hematoxylin and eosin stain)**. There are no significant epithelial atypia and no invasion into the deeper tissue.

The frozen section of this mass showed extensive foci of dystrophic calcification and non-specific acute and chronic inflammation. A detailed histopathology report of the appendix along with the attached mesoappendix showed a 1.5 cm perforation in the lumen of the appendix with mucopurulent exudate in the surrounding area. Mucinous material was observed in the dilated appendicular lumen. Tall columnar, hyperplastic mucosa with focal papillary architecture lining the appendix was identified. Foci of dystrophic calcification, extensive areas of fibrosis and patchy acute and chronic non-specific inflammation were also seen in the wall of the appendix. No significant cytological atypia or invasion of the appendiceal wall by atypical glands was noted (Figures 2 and 3). A final histopathological diagnosis of mucinous cystadenoma was made. The patient remained well post-operatively and was discharged after an uneventful hospital stay. At one-year follow-up, the patient was symptom-free.

## Discussion

Mucinous cystadenoma of the appendix can be asymptomatic or may present with abdominal pain, an abdominal mass, per rectal bleeding, ureteral obstruction, hematuria or intussusception [1,5-8]. Our patient had a 15-day history of pain in her RIF. She had also been experiencing some heaviness in the lower abdomen for six months. Although the presence of symptoms in a patient with an appendiceal mucocele has been reported to be associated with a higher incidence of cystadenocarcinomas [9], this does not appear to be a useful guide in the pre-operative assessment of patients with appendiceal mucoceles.

Colonoscopy and CT scan were used as the initial investigative modalities in our patient. CT scan findings in patients with mucinous cystadenomas include cystic masses with low attenuation, irregular wall thickening and absence of associated appendiceal inflammation [1,7]. Mural calcification has been reported with a high frequency (50%) in patients with appendicular mucinous cystadenomas [10]. It is also important to note that the varying thickness of the wall of the mass on CT scans has not been shown to correlate with malignancy. However, contrast enhancing nodules on CT scans may be suggestive of cystadenocarcinomas [1]. A colonoscopy performed on our patient revealed external compression of the cecum with normal overlying mucosa. We then proceeded with a CT scan which showed an appendicular mucocele with extensive mural calcification and some periappendiceal stranding.

Both mucinous cystadenomas and cystadenocarcinomas have the potential to cause peritoneal seeding leading to pseudomyxoma peritonei. However, survival in patients with a cystadenoma is better compared to patients with its malignant counterpart when considering pseudomyxoma peritonei [1,11,12].

It is important to distinguish between mucinous cystadenomas and mucinous cystadenocarcinomas. However, this distinction remains elusive and cannot be established with any degree of reliability if we are to depend solely on physical examination findings and radiological imagings. Histopathological examination is instrumental in achieving an accurate diagnosis. Herein, lies the dilemma for the surgeon because of the nebulous nature of the distinction between these entities in the pre-operative setting.

The best surgical management of a patient with an appendiceal mucocele remains a subject of controversy. There is little consensus on the optimal choice of procedure (right hemicolectomy versus appendectomy) as well as the approach (laparoscopic versus laparotomy). While earlier data have shown a survival advantage associated with right hemicolectomy, more recent prospective data findings do not report any survival advantages in a patient with pseudomyxoma peritonei syndrome and appendiceal mucinous carcinomatosis undergoing a right hemicolectomy [13-15].

In general, certain principles must be kept in mind while operating on patients with appendiceal mucoceles. It is important to exercise care in handling tissues intra-operatively to reduce the risk of dissemination of mucin-producing epithelium [1]. Zagrodnik *et al*. have supported the choice for appendectomy with mesoappendix excision in the absence of local invasion or cecal involvement for appendiceal masses [1]. Gupta *et al*. have advocated the removal of mucoceles of less than 2 cm in diameter using this approach [7]. However, mucoceles of less than 2 cm in diameter are usually simple retention cysts and this choice of surgical approach based on size alone does not appear to be helpful considering that hyperplastic epithelium, cystadenoma and cystadenocarcinoma are more likely to be greater than 2 cm in size [3].

The presence of local invasion and cecal involvement are two indications in the literature that necessitate employment of right hemicolectomy for appendiceal mucoceles [1]. An open approach has been favored by some authors as being a definitive and safe maneuver which allows better visualization of the abdominal cavity. This advocacy is supported by the incidence of peritoneal implants and inadvertently missed lesions after laparoscopy [1,9]. The right retrohepatic space, pelvis, omentum and left paracolic space all merit meticulous inspection for mucinous fluid collection. The appendiceal lymph nodes and appendiceal stump should also be carefully inspected [9].

## Conclusion

It would be prudent for surgeons to exercise clinical acumen in choosing the best procedure for patients in the absence of unequivocally reproducible prospective data. We want to highlight the value of using frozen sections in the surgical management for patients with appendicular mucoceles. This is a reasonable intra-operative approach to pursue in selected cases whereby the operation can be extended to include a right hemicolectomy if the findings so warrant. Lymph node status and margin positivity can serve as useful guides in this regard [9].

Our patient was symptom-free at one-year follow-up. Long-term follow-up of our patient will help to ascertain the safety profile of the approach we employed. Systematic work-up and follow-up of patients with mucinous cystadenoma of the appendix should be undertaken because of the reported association with ovarian mucinous cystoma and large bowel adenocarcinoma [6,8].

## Abbreviations

CT: computed tomography; RIF: right iliac fossa.

## Competing interests

The authors declare that they have no competing interests.

## Authors' contributions

TS collected the data, helped in its interpretation and drafted the manuscript. RA helped in identification and interpretation of pathology along with drafting the manuscript. MRK conceived the study, helped in the interpretation of the data, drafted the manuscript and provided overall supervision in the project. All authors read and approved the final manuscript.

## Consent

Written informed consent was obtained from the patient for publication of this case report and any accompanying images. A copy of the written consent is available for review by Editor-in-Chief of this journal.
